# Sparse Rectangular and Spiral Array Designs for 3D Medical Ultrasound Imaging

**DOI:** 10.3390/s20010173

**Published:** 2019-12-27

**Authors:** Hansol Yoon, Tai-Kyong Song

**Affiliations:** Department of Electronic Engineering, Sogang University, Seoul 04107, Korea; yoonhs@sogang.ac.kr

**Keywords:** sparse 2D array, 3D ultrasound imaging, rectangular array, spiral array, sunflower array, grating lobe suppression, array signal processing

## Abstract

In three-dimensional (3D) medical ultrasound imaging with two-dimensional (2D) arrays, sparse 2D arrays have been studied to reduce the number of active channels. Among them, sparse 2D arrays with regular or uniform arrangements of elements have advantages of low side lobe energy and uniform field responses over the entire field of view. This paper presents two uniform sparse array models: sparse rectangular arrays (SRAs) on a rectangular grid and sparse spiral arrays (SSAs) on a sunflower grid. Both arrays can be easily implemented on the commercially available or the custom-made arrays. To suppress the overall grating lobe levels, the transmit (Tx) and receive (Rx) array pairs of both the array models are designed not to have grating lobes at the same locations in the Tx/Rx beam patterns, for which the theoretical design rules are also proposed. Computer simulation results indicate that the proposed array pairs for both the SRAs and the SSAs achieve peak grating lobe levels below –40 dB using about a quarter of the number of elements in the dense rectangular array while maintaining similar beam widths to that of the dense array pair.

## 1. Introduction

Ultrasound two-dimensional (2D) array transducers provide a powerful means of fast and high-resolution three-dimensional (3D) ultrasound imaging. Theoretically, to avoid grating lobes, the interelement distance of a 2D array should be limited to half the acoustic wavelength. Consequently, dense 2D arrays usually have a large number of elements, causing a tremendous increase in the computational or the hardware complexity for ultrasound beamforming. Such huge computational costs can also hinder dense 2D arrays from realizing 3D imaging with desired volume rates and spatial resolutions. Thus, reducing the active elements used for real-time 3D imaging has been a very important research topic in 2D array imaging.

Sparse 2D arrays are one of the strategies to reduce the number of active elements by undersampling dense arrays. However, because sparse arrays with regularly spaced active elements cause the elevation of grating lobes, various methods to design transmit and receive (Tx/Rx) sparse array pairs with reduced grating lobe levels have been suggested. Placing elements irregularly is a reasonable approach to spread the concentrated grating lobe energy of a regular sparse array over the entire acoustic field [1]. This approach has been improved by using optimization theories [2,3,4]. In search of more reliable and computationally efficient sparse arrays, an analytical method utilizing the so-called almost difference sets (ADS) was also proposed [5].

However, due to the non-uniform distribution of the elements, all the methods mentioned above have several problems: the difficulty of guaranteeing the uniformity among the scan lines; restriction of the element size, and the increase in the overall side lobe energy. To overcome these problems, sparse periodic array layout schemes, such as Vernier arrays, have been proposed [6,7,8,9]. Vernier arrays have non-overlapping grating lobe patterns which result in overall two-way beam patterns with suppressed grating lobes. Recently, our group presented an analytical model for generalized sparse periodic linear arrays [10]. Based on the analytical model, a design rule to avoid common grating lobes of Tx/Rx 1D array pairs was developed.

Several sparse array layouts for non-rectangular 2D arrays have also been proposed, such as concentric circular arrays [1,11,12] and spiral arrays [13,14,15]. The sunflower array, which is a special case of Fermat’s spiral array, is known to have advantages over the other sparse array designs in terms of beamforming performance and array uniformity. However, the design criteria and methods for optimal Tx/Rx spiral arrays are not yet established. In a previous study [14], for example, the rotation angle between Tx/Rx Fermat’s spiral arrays was optimized, but the possibility of the overlapping Tx/Rx elements in one probe was not considered. Practically, this will bring manufacturing problems.

This paper expands the previous sparse linear array (SLA) design method [10] for designing sparse rectangular arrays (SRAs) with elements placed on a rectangular grid and sparse spiral arrays (SSAs) having elements on a sunflower grid. First, specific configurations for each of the generalized SRA and SSA are suggested. Then, the theoretical models for the field responses of the SRAs and the SSAs are presented to assess the important beamforming performances, such as main lobe beam width and grating lobe patterns. Based on the analysis results, simple design rules for the Tx/Rx pairs of both the SRAs and the SSAs are proposed. The design rules pair the Tx/Rx arrays in a way that the arrays do not produce grating lobes at the same locations. Finally, the array pairs are evaluated and compared through computer simulations, showing that the proposed design rules for both array schemes are valid.

## 2. Sparse Rectangular Array

### 2.1. Array Model

**Figure 1:**
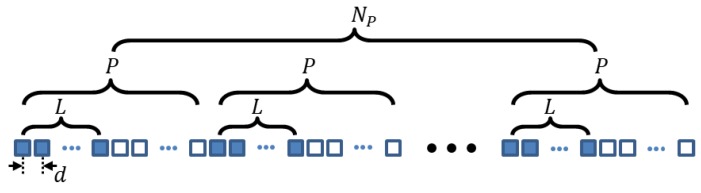
Sparse linear array layout. The array comprises *N_P_* blocks. In each block having *P* elements, only the subarray consisting of *L* consecutive elements (blue colored squares) in each block is active.

In our previous study, a design rule for eliminating common grating lobes of a pair of Tx/Rx arrays, each array having the form of the SLA model shown in Figure 1, was presented [10]. In this model, *L* consecutive elements are active in each *P*-element block, where *P* is greater than *L*. The *L* active elements are defined to be a subarray, and each block containing the subarray is repeated *N_P_* times to form the SLA model. Thus, in an SLA, only *N_P_*∙*L* elements are active among *N_P_*∙*P* elements.

**Figure 2:**
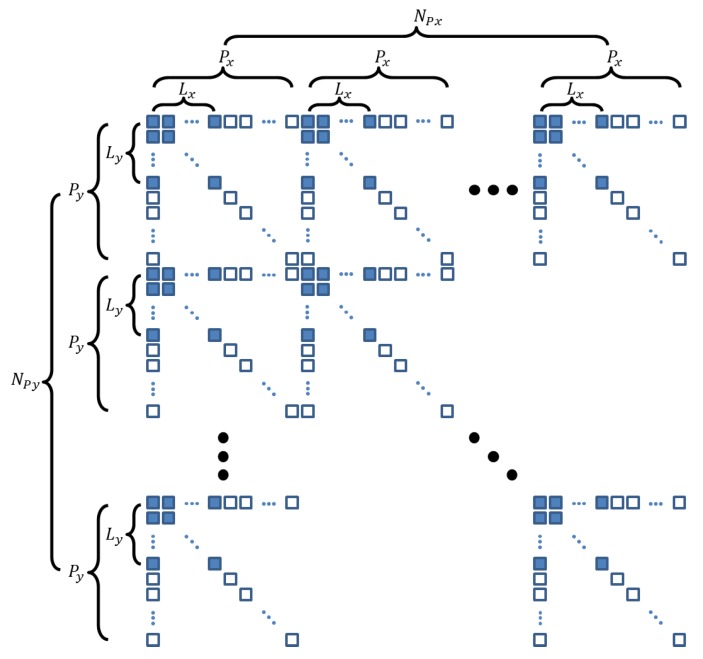
Sparse rectangular array layout. The array comprises *N_Px_*∙*N_Py_* blocks. In each block having *P_x_*∙*P_y_* elements, only the two-dimensional (2D) subarray consisting of *L_x_*∙*L_y_* elements (blue colored squares) is active.

Here we generalize the SLA model to the SRA model, as shown in Figure 2. Generally, an SRA comprises *N_Px_*∙*N_Py_* blocks. Each block is composed of *P_x_*∙*P_y_* elements, and in each block, only the subarray consisting of *L_x_*∙*L_y_* elements is active. Such SRA will be denoted as SRA-*L_x_L_y_* for convenience.

**Figure 3:**
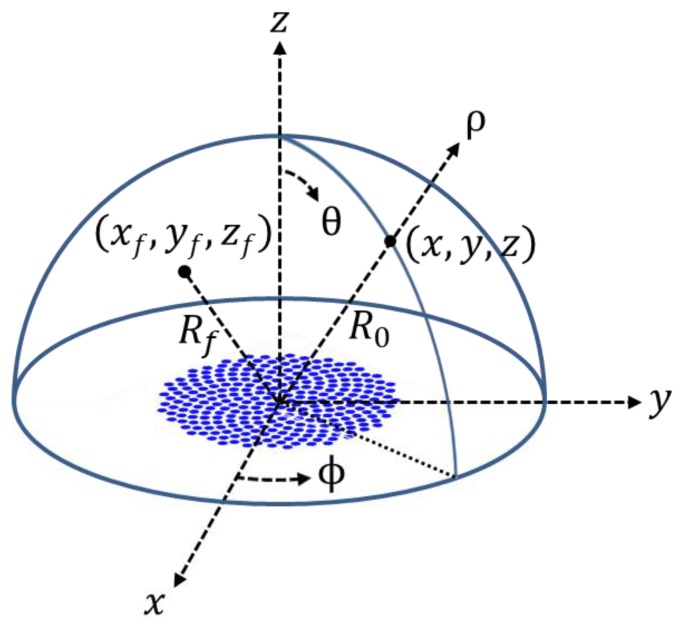
Coordinate system for the theoretical analysis and the beam simulations.

Using the parameters in Figure 2 and the coordinates in Figure 3, the far-field, continuous wave (CW) response of an SRA, Ψ_SRA-*LxLy*_(*x*,*y*,*z*), can be approximately expressed in the spatial domain as

(1) $$\Psi_{{\mathrm{SRA}-L}_{x}L_{y}}\left(x,y,z\right)\;=\;\mathrm{sinc}\left(w_{x}\frac{x}{{\lambda R}_{0}}\right)\;\cdot \overset{N_{Px}-1}{\underset{m_{x}=0}{\sum }}\overset{L_{x}-1}{\underset{n_{x}=0}{\sum }}\exp\left[-j\frac{2\pi }{\lambda }\left\{\left(\frac{x}{R_{0}}-\frac{x_{f}}{R_{f}}\right)P_{x}m_{x}{\;+\;n}_{x}\right\}d_{x}\right]\;\;\;\;\;\;\;\;\;\cdot \;\mathrm{sinc}\left(w_{y}\frac{y}{{\lambda R}_{0}}\right)\;\cdot \overset{N_{Py}-1}{\underset{m_{y}=0}{\sum }}\overset{L_{y}-1}{\underset{n_{y}=0}{\sum }}\exp\left[-j\frac{2\pi }{\lambda }\left\{\left(\frac{y}{R_{0}}-\frac{y_{f}}{R_{f}}\right)P_{y}m_{y}{\;+\;n}_{y}\right\}d_{y}\right],$$

where (*x_f_,y_f_,z_f_*) is the far-depth focus, λ is the wavelength, *w_x_* and *w_y_* are the element widths, *d_x_* and *d_y_* are the element pitches, *R*_0_ is the distance between the observation point and the array center, and *R*_f_ is the distance between the focal point and the array center [10,16].

By using the spherical coordinates *u_x_* = *u_x_*_0_ − *u_x_*_f_ = *x*/*R*_0_ − *x*_f_/*R*_f_ and *u_y_* = *u_y_*_0_ − *u_y_*_f_ = *y*/*R*_0_ − *y*_f_/*R*_f_, Equation (1) can be rewritten as

(2) $$\Psi_{{\mathrm{SRA}-L}_{x}L_{y}}\left(u_{x}{,u}_{y}\right)\;=\;\mathrm{sinc}\left(w_{x}\frac{u_{x0}}{\lambda }\right)\;\cdot \overset{N_{Px}-1}{\underset{m_{x}=0}{\sum }}\overset{L_{x}-1}{\underset{n_{x}=0}{\sum }}\exp\left\{-j\frac{2\pi }{\lambda }\left(u_{x}P_{x}m_{x}{\;+\;n}_{x}\right)d_{x}\right\}\;\;\;\;\;\;\;\;\;\;\cdot \;\mathrm{sinc}\left(w_{y}\frac{u_{y0}}{\lambda }\right)\;\cdot \overset{N_{Py}-1}{\underset{m_{y}=0}{\sum }}\overset{L_{y}-1}{\underset{n_{y}=0}{\sum }}\exp\left\{-j\frac{2\pi }{\lambda }\left(u_{y}P_{y}m_{y}{\;+\;n}_{y}\right)d_{y}\right\}.$$

Because *x* and *y* terms of Ψ_SRA-*LxLy*_(*u_x_*,*u_y_*) are separable, Equation (2) can be expressed as

(3) $$\Psi_{{\mathrm{SRA}-L}_{x}L_{y}}\left(u_{x}{,u}_{y}\right){\;=\;\Psi }_{{\mathrm{SLA}-L}_{x}}\left(u_{x}\right)\;\cdot {\;\Psi }_{{\mathrm{SLA}-L}_{y}}\left(u_{y}\right),$$

where, in fact, Ψ_SLA-*Lx*_(*u_x_*) and Ψ_SLA-*Ly*_(*u_y_*) are the far-field, CW responses of the SLAs in the *x-* and *y-* axes, respectively.

Furthermore, by solving the double summation of the exponentials in Equation (2), Ψ_SLA-*Lx*_(*u_x_*) in Equation (3) can be further factorized into the element factor Φ_e_(*u_x_*_0_), the basic SLA factor Ψ_SLA-1_(*u_x_*) (i.e., the array factor of an SLA with *L* = 1) and the subarray factor Ψ_SA-*L*_(*u_x_*) [10]. That is,

(4) $$\Psi_{{\mathrm{SLA}-L}_{x}}\left(u_{x}\right)\propto {\;\Phi }_{e}\left(u_{x0}\right)\;\cdot {\;\Psi }_{\mathrm{SLA}-1}\left(u_{x}\right)\;\cdot {\;\Psi }_{{\mathrm{SA}-L}_{x}}\left(u_{x}\right),$$

where

(5) $$\Phi_{e}\left(u_{x0}\right)\;=\;\mathrm{sinc}\left(w\frac{u_{x0}}{\lambda }\right),$$

and

(6) $$\Psi_{\mathrm{SLA}-1}\left(u_{x}\right)\;=\;\frac{\sin\left({\pi N}_{P}{Pdu}_{x}/\lambda \right)}{\sin\left({\pi Pdu}_{x}/\lambda \right)},$$

and

(7) $$\Psi_{{\mathrm{SA}-L}_{x}}\left(u_{x}\right)\;=\;\frac{\sin\left({\pi dLu}_{x}/\lambda \right)}{\sin\left({\pi du}_{x}/\lambda \right)}.$$

The *y* term of Equation (3), Ψ_SLA-*Ly*_(*u_y_*), can also be factorized in the same way as Ψ_SLA-*Lx*_(*u_x_*) (4). Therefore, by incorporating all the factors (4)–(7) into Equation (3), the far-field, CW response of an SRA can be represented as

(8) $$\Psi_{{\mathrm{SRA}-L}_{x}L_{y}}\left(u_{x}{,u}_{y}\right)\propto {\;\Phi }_{e}\left(u_{x0}{,u}_{y0}\right)\;\cdot {\;\Psi }_{\mathrm{SLA}-1}\left(u_{x}\right)\;\cdot {\;\Psi }_{{\mathrm{SA}-L}_{x}}\left(u_{x}\right)\;\cdot {\;\Psi }_{\mathrm{SLA}-1}\left(u_{y}\right)\;\cdot {\;\Psi }_{{\mathrm{SA}-L}_{y}}\left(u_{y}\right),$$

where Φ_e_(*u_x_*_0_,*u_y_*_0_) = Φ_e_(*u_x_*_0_) ∙ Φ_e_(*u_y_*_0_) represents the 2D element factor.

To understand the characteristics of the beam pattern of Ψ_SRA-*LxLy*_(*u_x_*,*u_y_*), it is instructive to evaluate the main lobe width and the grating lobe locations of the basic SRA factor Ψ_SRA-1_(*u_x_*,*u_y_*) = Ψ_SLA-1_(*u_x_*) ∙ Ψ_SLA-1_(*u_y_*) that represents Ψ_SRA-*LxLy*_(*u_x_*,*u_y_*) when *L_x_* = *L_y_* = 1. The grating lobe locations of Ψ_SRA-1_(*u_x_*,*u_y_*) are calculated from Equation (6) as

(9) $$\left(u_{x}{,u}_{y}\right)\;=\;{\left(u_{x}{,u}_{y}\right)}_{GL}\;=\;\left(\frac{\lambda }{P_{x}d}\cdot m,\frac{\lambda }{P_{y}d}\cdot n\right),\;\left(m,n\right)\left\{{\mathbb{Z}}^{2}-\left(0,0\right)\right\}.$$

In addition, the null-to-null main lobe width of Ψ_SRA-1_(*u_x_*,*u_y_*) is 2λ/(*N_P_Pd)*, which is equivalent to that of a dense array with *N_Px_*∙*P_x_*∙*N_Py_*∙*P_y_* elements. Now, except for the element factor, two remaining factors of Ψ_SRA-*LxLy*_(*u_x_*,*u_y_*) (8) can be treated as a 2D subarray factor (a small dense 2D array), Ψ_SA-*LxLy*_(*u_x_*,*u_y_*) = Ψ_SA-*Lx*_(*u_x_*) ∙ Ψ_SA-*Ly*_(*u_y_*). From Equation (7), it is easy to show that the factor Ψ_SA*-LxLy*_(*u_x_*,*u_y_*) has a null grid of

(10) $$u_{x}{\;=\;u}_{x,\mathrm{null}}\;=\;\frac{\lambda }{L_{x}d}\cdot {m,\;u}_{y}{\;=\;u}_{y,\mathrm{null}}\;=\;\frac{\lambda }{L_{y}d}\cdot n,\;\left(m,n\right)\left\{{\mathbb{Z}}^{2}-\left(0,0\right)\right\}.$$

A null grid is made up of vertical (*u_x_* = *u_x_*_,null_) and horizontal (*u_y_* = *u_y_*_,null_) lines, and ideally, the field response is zero on the lines. Thus, the grating lobe positions (9) that are located on the null grid (10) will vanish. In fact, by choosing the values of *L_x_* and *L_y_* properly, one can eliminate unwanted grating lobes in specific locations. For an extreme case, if *P_x_* = *L_x_* and *P_y_* = *L_y_*, which is the dense array case, every grating lobe position is on the null grid, and hence, such an array does not have any grating lobes.

### 2.2. Design Rule

The Tx/Rx rectangular array pair (TRA/RRA) will be represented by their values of *P* and *L* as TRA(*P_x,_*_T_∙*P_y,_*_T_, *L_x,_*_T_∙*L_y,_*_T_)/RRA(*P_x,_*_R_∙*P_y,_*_R_, *L_x,_*_R_∙*L_y,_*_R_). Surely, the rectangular array is dense if *P_x_*∙*P_y_* = *L_x_*∙*L_y_* and sparse if *P_x_*∙*P_y_* > *L_x_*∙*L_y_*. In the previous subsection, it has been shown that the grating lobe positions of the SRAs are governed by the grating lobe position (9) and the null grid (10). Now the common grating lobes (CGLs) of the TRA/RRA pairs are investigated. The CGLs represent the grating lobes that appear at the same locations in both the Tx/Rx beam patterns. Because a TRA/RRA pair with CGLs will produce strong grating lobes in its round-trip response, it must be designed not to have CGLs.

In the SLA case [10], the CGL positions of an SLA pair were given by

(11) $$u\;=\;\frac{m}{{\mathrm{GCD}(P}_{T}{,P}_{R})}\cdot \frac{\lambda }{d},\;m\;\{\mathbb{Z}-(0)\},$$

where GCD(∙) is the greatest common divider of the two arguments. Since the SRA is the 2D generalization of the SLA, the CGL positions of an SRA pair are calculated as

(12) $$\left(u_{x}{,u}_{y}\right)\;=\;{\left(u_{x}{,u}_{y}\right)}_{CGL}=\;\left(\frac{m}{\mathrm{GCD}\left(P_{x,T}{,P}_{x,R}\right)}\cdot \frac{\lambda }{d},\frac{n}{\mathrm{GCD}\left(P_{y,T}{,P}_{y,R}\right)}\cdot \frac{\lambda }{d}\right),\;\left(m,n\right)\left\{{\mathbb{Z}}^{2}-\left(0,0\right)\right\}.$$

The essential rule for designing optimal TRA/RRA pairs is to suppress the CGLs, if they exist, by choosing *L_x_*_,R_ and *L_y_*_,R_ as

(13) $$L_{x,R}{\;=\;k}_{x}\cdot \mathrm{GCD}\left(P_{x,T}{,P}_{x,R}\right),$$

and

(14) $$L_{y,R}{\;=\;k}_{y}\cdot \mathrm{GCD}\left(P_{y,T}{,P}_{y,R}\right),$$

where *k_x_* and *k_y_* are natural numbers that do not make *L_x_*_,R_ and *L_y_*_,R_ exceed *P_x_*_,R_ and *P_y_*_,R_, respectively. Note that the CGL positions (12) locate perfectly on the null grid (10) with the use of the design rule (13) and (14), which will result in canceling all the CGLs of the array pair. For the transmit array, *L_x_*_,T_ and *L_y_*_,T_ can be any natural numbers not greater than *P_x_*_,T_ and *P_y_*_,T,_ respectively, as long as the CGL-free conditions (13) and (14) are met. One can also make *L_x_*_,T_ and *L_y_*_,T_ meet the CGL-free conditions and choose *L_x_*_,R_ and *L_y_*_,R_ freely.

## 3. Sparse Spiral Array

### 3.1. Array Model

Fermat’s spiral pattern is defined in polar coordinates as

(15) $$e_{n}\;=\;\left(l_{0}\sqrt{n\alpha },\;n\alpha \right){,\;n\;=\;1,\;\ldots ,\;N}_{e},$$

where *α* is the divergence angle, *N_e_* is the number of element points, and *l*_0_ is defined to achieve the desired aperture diameter *D* of the array as

(16) $$l_{0}\;=\;\frac{D}{2\sqrt{\left(N_{e}-1\right)\alpha }}.$$

In this paper, the sunflower pattern, which is a special case of the Fermat’s spiral pattern (15) where *α* = *α*_0_ = 137.51°, is chosen as the dense spiral array grid. Sunflower arrays are known for their good element packing property and having beam patterns with low side lobe energy [14,15]. In addition, it is advantageous to choose the active elements out of the sunflower array since the sunflower pattern is the densest among the spiral patterns.

The element centers of an SSA with *N_s_* elements (*N_s_* = *N_P__α_*∙*L_α_* ≤ *N_e_*) are suggested to be defined in the sunflower grid as

(17) $$s_{n}\;=\;\left(l_{n}{\;=\;l}_{0}\sqrt{\alpha_{n}}{,\;\alpha }_{n}\right){,\;n\;=\;1,\;\ldots ,\;N}_{s},$$

where

(18) $$\alpha_{n}{\;=\;n\alpha }_{0}\;=\;\{(p-{1)P}_{\alpha }{\;+\;q\}\alpha }_{0},\;1\;\leq \;p\;\leq {\;N}_{P\alpha },\;1\;\leq \;q\;\leq {\;L}_{\alpha }.$$

**Figure 4:**
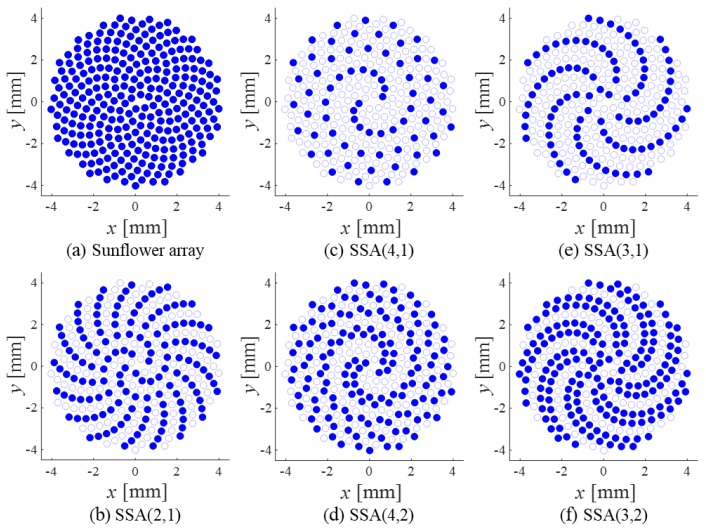
Examples of the spiral arrays: (**a**) the sunflower array and (**b**–**f**) the SSAs. Active elements are colored in blue.

Note that the angular positions {*α_n_*} in Equation (18) can be illustrated with the active elements of the SLA shown in Figure 1 with *d*, *P*, *L*, and *N_P_* replaced by *α*_0_, *P_α_*, *L_α_*, and *N_Pα_*, respectively. Figure 4 shows some examples of the SSAs denoted by SSA(*P_α_*, *L_α_*): (a) the sunflower array, (b) SSA(2,1), (c) SSA(4,1), (d) SSA(4,2), (e) SSA(3,1), and (f) SSA(3,2). Indeed, as shown in Figure 4, each of the SSAs defined by the polar coordinates (17) is the spiral version of the corresponding SLA defined by Equation (18). Given the exact locations (17) of the elements, the far-field, CW response of an SSA can be approximately expressed as

(19) $$\Psi_{\mathrm{SSA}}\left(u_{x}{,u}_{y}\right)\propto {\;\Phi }_{e}\left(u_{x0}{,u}_{y0}\right)\;\cdot \overset{N_{s}-1}{\underset{n=0}{\sum }}\exp\left[-j\frac{2\pi }{\lambda }\left\{u_{x}l_{n}\cos\left(\alpha_{n}\right){\;+\;u}_{y}l_{n}\sin\left(\alpha_{n}\right)\right\}\right],$$

which, unlike that of the SRA, cannot be factorized further. Nonetheless, the grating globe positions of the SSAs can be investigated.

It has been previously shown that the sunflower arrays are expected to produce circular symmetric grating lobe patterns, namely grating lobe rings [17]. In addition, the radii of the grating lobe rings are approximately given by integer multiples of $\lambda /\hat{d}$, where $\hat{d}$, the most likely interelement distance, can be calculated from the histogram of the lengths of the Delaunay-triangulation line segments of the sunflower array [17,18]. Therefore, because the SSAs are designed from the same model of the SLAs, the grating lobe rings of the SSAs when *L_α_* = 1 are expected to be at

(20) $$r=r_{gl}=\frac{\lambda }{P_{\alpha }\hat{d}}\cdot m,m\in \{\mathbb{Z}-\left(0\right)\},$$

where $r^{2}{\;=\;u}_{x}{}^{2}{\;+\;u}_{y}{}^{2}$. Note that, by setting *P_α_* = 1, the grating lobe rings of the sunflower arrays can also be explained by Equation (20).

In addition, when *L_α_* > 1 for a given value of *P_α_*, the grating lobes at the following locations are expected to be suppressed.

(21) $$r=r_{\mathrm{null}}=\frac{\lambda }{P_{\alpha }\hat{d}}\cdot m,m\in \{\mathbb{Z}-\left(0\right)\}.$$

Analogous to the null grid for SRAs, we shall assume that there exist low amplitude rings with the radii calculated by Equation (21), which will be called the nulling rings for convenience.

### 3.2. Design Rule

As the element centers of an SSA are sparsely selected from a sunflower grid according to Equations (17) and (18) depending on the values of *P_α_* and *L_α_*, the Tx/Rx SSA (TSA/RSA) pair will be represented by TSA(*P_α_*_,T_*,L_α_*_,T_)/RSA(*P_α_*_,R_*,L_α_*_,R_). Because the radii of the grating lobe (20) and the nulling rings (21) of an SSA are theoretically given, one can easily find that the CGL positions of an SSA pair are derived as

(22) $$r\;=\;\frac{m}{{\mathrm{GCD}(P}_{\alpha ,T}{,P}_{\alpha ,R})}\cdot \frac{\lambda }{d},\;m\;\{\mathbb{Z}-(0)\},$$

which resembles the SLA case (11). Thus, the SSA pairs will not have CGLs when the same design rule for the CGL-free SLA pairs is adopted as

(23) $$L_{\alpha ,R}\;=\;k\cdot \mathrm{GCD}\left(P_{\alpha ,T}{,P}_{\alpha ,R}\right).$$

The value of *L_α_*_,T_ can be any natural number not greater than *P_α_*_,T_ just as in the SRA case. The validation of this design rule is demonstrated in the following section.

## 4. Simulations Results

### 4.1. Simulation Environment

Both the far-field, CW field response and the near-field, pulsed wave (PW) response were calculated on a constant-ρ hemispherical surface shown in Figure 3. The coordinate of a point lying on the hemispherical surface is represented by (*u_x_*_0_, *u_y_*_0_) = (sinθcosφ, sinθsinφ) in the range of −90° ≤ θ ≤ 90° and −90° ≤ φ ≤ 90°. The field responses were investigated for two cases: (*u_x_*_f_, *u_y_*_f_) = (0, 0) and (0.5,0) (i.e., θ_f_ = π/6 and φ_f_ = 0), where (*u_x_*_f_, *u_y_*_f_) denotes the focal point on the same hemispherical surface. We remind that the coordinates in Ψ_SRA-*LxLy*_(*u_x_*,*u_y_*) and Ψ_SSA_(*u_x_*,*u_y_*) in the previous sections are expressed as (*u_x_*, *u_y_*) = (*u_x_*_0_ − *u_x_*_f_, *u_y_*_0_ − *u_y_*_f_).

The CW responses were calculated from the theoretical models (8,19) presented in Section 2 and Section 3, and Field II program [19,20] was used to obtain the PW responses. The following parameters were used in the simulations for both SRAs and SSAs: sound speed of *c* = 1540 m/s; central frequency of *f_0_* = 3 MHz; sampling frequency of *f_s_* = 100 MHz; focal depth of *F* = 40 mm; transducer impulse response equal to a two-cycle sine pulse with a Hanning window; and a two-cycle sine excitation.

### 4.2. Evaluation Metrics

The peak grating lobe level (PGL) and the main lobe-to-side lobe energy ratio (MSR) are known to be good performance measures for the beamforming arrays [1,7], which are calculated as

(24) $$\mathrm{PGL}\;=\;\frac{\underset{u_{x}^{2}{+u}_{y}^{2}\geq 0.2^{2}}{\max}{\left|\mathrm{PSF}\left(u_{x}{,u}_{y}\right)\right|}^{2}}{{\left|\mathrm{PSF}\left(0,0\right)\right|}^{2}},$$

and

(25) $$\mathrm{MSR}\;=\;\frac{\sum^{\;}\sum^{\;}{\left|\mathrm{PSF}\left(u_{x}{,u}_{y}\right)\right|}^{2}\mathrm{ML}\left(u_{x}{,u}_{y}\right)}{\sum^{\;}\sum^{\;}{\left|\mathrm{PSF}\left(u_{x}{,u}_{y}\right)\right|}^{2}\left\{1-\mathrm{ML}\left(u_{x}{,u}_{y}\right)\right\}},$$

where PSF(*u_x_,u_y_*) is the point spread function of an array. In Equation (25), ML(*u_x_,u_y_*) is a windowing function which value is one in the main lobe region restricted by −50 dB beam width (BW) and 0 outside the main lobe region. The decision boundaries of the PGL and the main lobe region were adopted from [5,8]. In addition to the PGL and MSR values, the BWs of the main lobe at −6 dB (BW_6_) and −50 dB (BW_50_) were measured.

### 4.3. Sparse Rectangular Array

**Figure 5:**
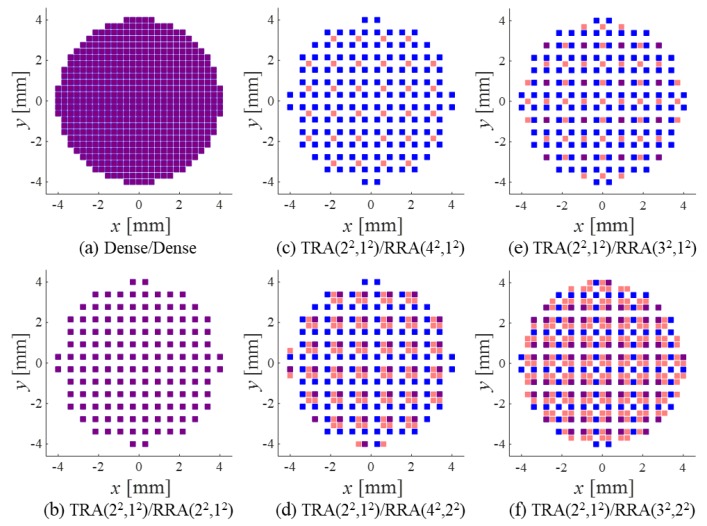
Transmit (blue) and receive (red) rectangular array pairs: (**a**) the dense array pair and (**b**–**f**) the sparse rectangular array (SRA) pairs. The overlapping elements are colored in purple.

The Tx/Rx array pairs for the beam simulations are shown in Figure 5. A circular window with a diameter of 16λ was used to reduce the weak-contributing elements in the edges of the array model in Figure 2 [21]. Square-shaped elements with the element width of 0.5λ and the element pitch of 0.6λ (both in *x* and *y* directions) were used. Ideally, if the element pitch was not greater than 0.5λ, then the grating lobes were not produced even when the focal point was steered. In this simulation, however, the element pitch (0.6λ) was chosen to be the same as that of a commercially available 2D array [4], which will be used in a future experimental study.

Figure 5a shows the dense array pair on a 27 × 27 grid whose beam pattern will serve as the reference for the evaluation of beamforming performance of sparse arrays. For the sparse array pairs in Figure 5b–f, the same TRA with the parameters of *P_x,_*_T_∙*P_y,_*_T_ = 2∙2 = 2^2^ and *L_x,_*_T_∙*L_y,_*_T_ = 1∙1 = 1^2^, denoted by TRA(2^2^,1^2^), was used, but the RRAs were chosen differently. The ones that follow the proposed design rule (Figure 5b,c) used the Rx arrays of RRA(2^2^,1^2^) and RRA(4^2^,1^2^), and the others that did not follow the rule (Figure 5d–f)) used the Rx arrays of RRA(4^2^,2^2^), RRA(3^2^,1^2^), and RRA(3^2^,2^2^).

**Figure 6:**
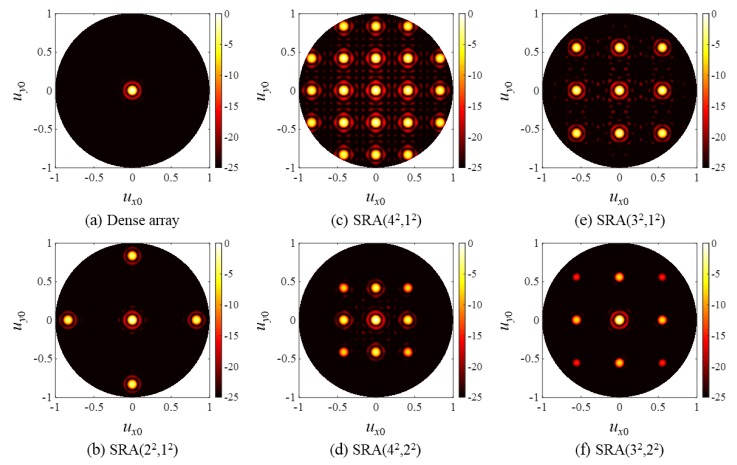
One-way, continuous wave (CW) responses of (**a**) the dense array and (**b**–**f**) the SRAs.

Figure 6 shows the one-way CW responses of the Rx arrays in Figure 5 when (*u_x_*_f_, *u_y_*_f_) = (0, 0). Thus, in this case, (*u_x_*, *u_y_*) = (*u_x_*_0_, *u_y_*_0_). The beam pattern of a dense array (Figure 6a) had no grating lobe artifacts and only the main lobe was observed. In the case of SRAs with *L_x,_*_R_ = *L_y,_*_R_ = 1 (Figure 6b,c,e), grating lobes were observed exactly at the locations predicted by Equation (9); the spacing between adjacent grating lobes in each of *x* and *y* directions decreased with increasing *P_x,_*_R_. When *L_x,_*_R_ = *L_y,_*_R_ = 2, Figure 6d,f show that the grating lobe artifacts are reduced. Especially for RRA(4^2^,2^2^) (Figure 6d), because of the 2D subarray factor Ψ_SA-2_(*u_x_*) ∙ Ψ_SA-2_(*u_y_*), the outer grating lobes of RRA(4^2^,1^2^) (Figure 6c) located on the null grid of *u_x_* = ±λ/2*d* and *u_y_* = ±λ/2*d* defined by Equation (10) were eliminated.

On the other hand, one can see by comparing Figure 6e,f that the grating lobes of RRA(3^2^,1^2^) were not completely eliminated in the response of RRA(3^2^,2^2^) since none of them fall onto the null grid of Ψ_SA-2_(*u_x_*) ∙ Ψ_SA-2_(*u_y_*). Although, because the CW response of RRA(3^2^,2^2^) was that of RRA(3^2^,1^2^) multiplied by the subarray factor Ψ_SA-2_(*u_x_*) ∙ Ψ_SA-2_(*u_y_*), its grating lobes in Figure 6f were smaller than those of RRA(3^2^,1^2^) shown in Figure 6e.

**Figure 7:**
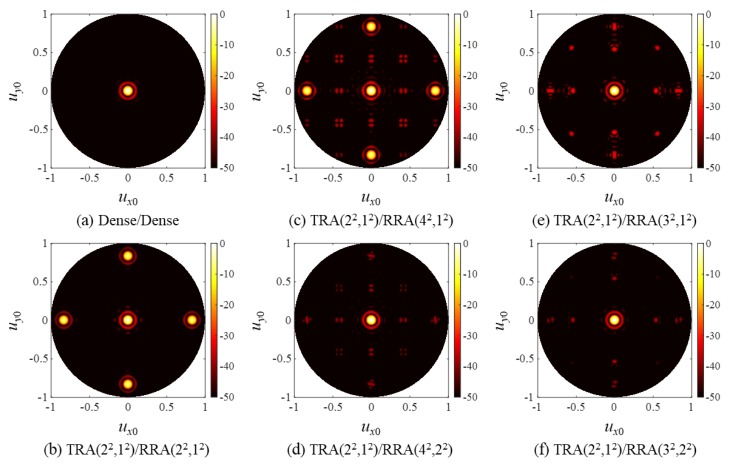
Two-way, CW point spread functions (PSFs) of (**a**) the dense array pair and (**b**–**f**) the SRA pairs.

The two-way CW PSFs of the array pairs were calculated by multiplying the Tx/Rx beam patterns. The results are shown in Figure 7. Note that TRA(2^2^,1^2^) was used on the transmission for all the SRA pairs, and its beam pattern was identical to that of RRA(2^2^,1^2^) in Figure 6b. According to Equation (12), all grating lobes of RRA(2^2^,1^2^) were CGLs when paired with TRA(2^2^,1^2^), as shown in the PSF of TRA(2^2^,1^2^)/RRA(2^2^,1^2^) in Figure 7b. Because GCD(4,2) = 2, the TRA(2^2^,1^2^)/RRA(4^2^,1^2^) pair also had CGLs at (*u_x_*, *u_y_*) = (±λ/2*d*, 0) and (*u_x_*, *u_y_*) = (0, ±λ/2*d*), as can be observed in Figure 7c. On the other hand, TRA(2^2^,1^2^)/RRA(4^2^,2^2^), TRA(2^2^,1^2^)/RRA(3^2^,1^2^), and TRA(2^2^,1^2^)/RRA(3^2^,2^2^) were CGL-free pairs satisfying the design rule (13) and (14), and thus high grating lobes are not found in Figure 7d–f.

**Figure 8:**
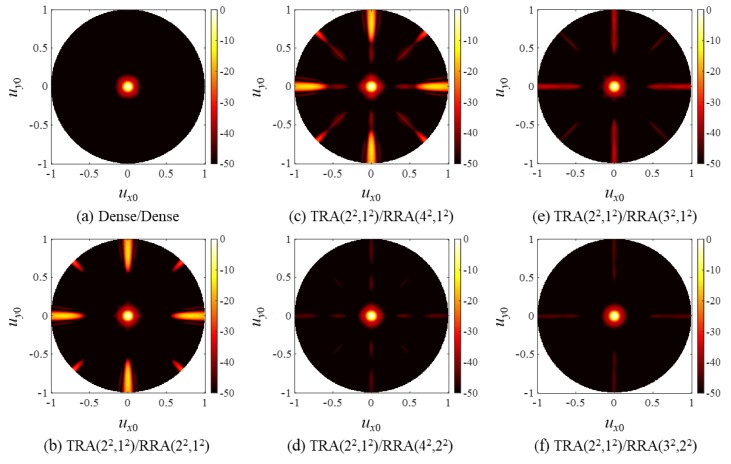
Two-way, pulsed wave (PW) PSFs of (**a**) the dense array pair and (**b**–**f**) the SRA pairs.

Figure 7 clearly shows that the CGL-free SRA pairs outperformed the SRA pairs that did not follow the design rule. Notably, the CGL-free TRA(2^2^,1^2^)/RRA(4^2^,2^2^) produced much smaller grating lobes than TRA(2^2^,1^2^)/RRA(2^2^,1^2^) using the same number of active elements. The same result was also observed in the PW PSFs in Figure 8, which have much broader and lower grating lobes than the corresponding CW PSFs due to the well-known property of wide band beamforming [22].

**Figure 9:**
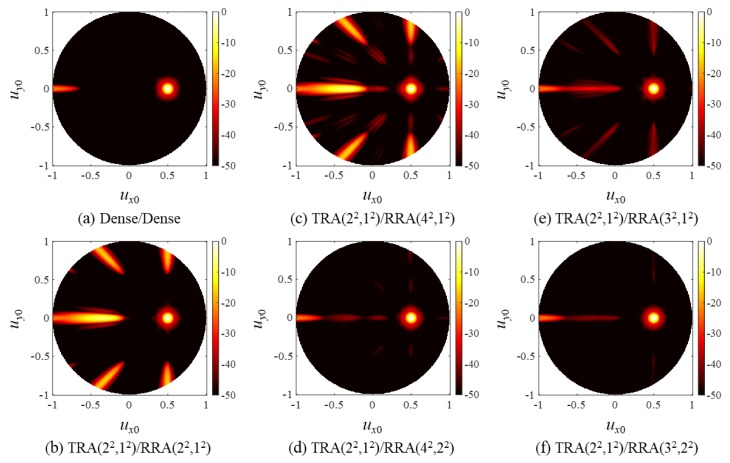
PW PSFs of (**a**) the dense array pair and (**b**–**f**) the SRA pairs for a steered focal point at (*u_x_*_f_,*u_y_*_f_) = (0.5,0) (i.e., θ_f_ = π/6, and φ_f_ = 0).

Figure 9 shows the steered PW PSFs of the SRA pairs. Note that all the PSFs of the dense array (Figure 9a) and the CGL-free SRA pairs (Figure 9d–f)) exhibited grating lobes in the vicinity of *u_x_*_0_ = −1 (i.e., θ = −π/2), which was not seen in the corresponding unsteered PSFs in Figure 8. It should be noted that the grating lobes near *u_x_*_0_ = −1 will not be generated if the element pitch is not greater than 0.5λ. Other grating lobes closer to the main lobe were surely suppressed as in the unsteered PSFs of the CGL-free SRA pairs in Figure 8.

**Figure 10:**
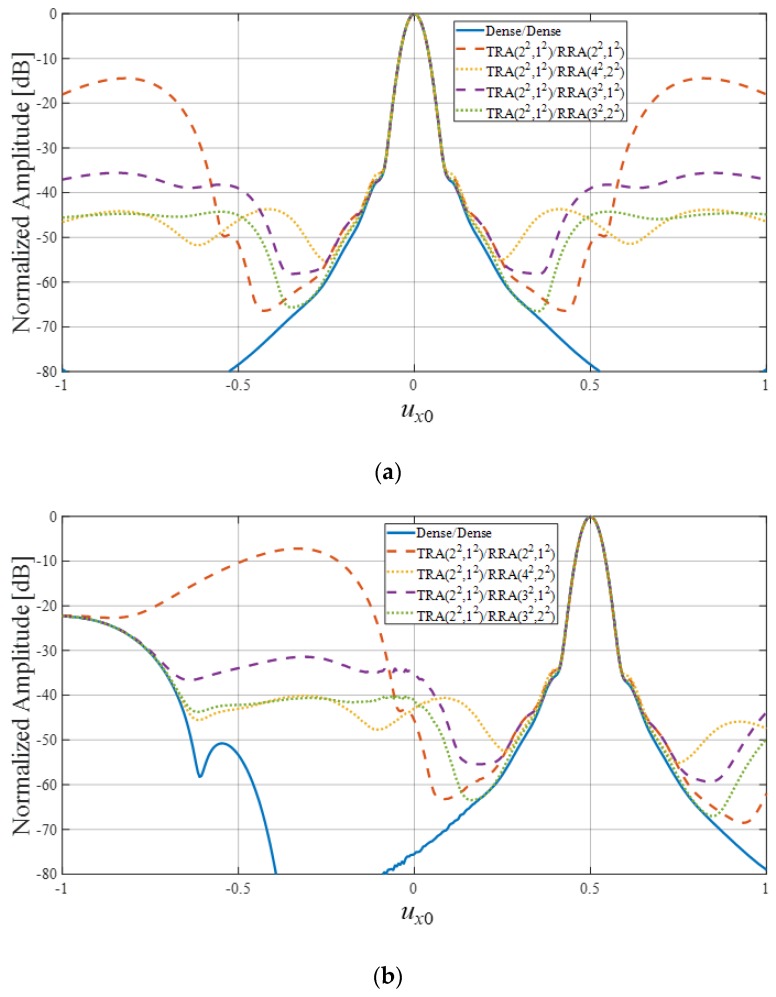
One-dimensional (1D) profiles (*u_y_*_0_ = 0) of (**a**) non-steered and (**b**) steered PW PSFs of the rectangular array pairs. The steered focal point was set to (*u_x_*_f_,*u_y_*_f_) = (0.5,0) (i.e., θ_f_ = π/6, and φ_f_ = 0).

To compare the non-steered, and the steered PW PSFs of the array pairs, their 1D profiles on the *u_x_*_0_ axis (*u_y_*_0_ = 0) are plotted in Figure 10. The low-performance array pair TRA(2^2^,1^2^)/RRA(4^2^,1^2^) was excluded for comparison. Figure 10 shows that all SRA pairs had almost the same BW_6_. However, TRA(2^2^,1^2^)/RRA(2^2^,1^2^) had significantly higher grating lobes than the other CGL-free SRA pairs.

For quantitative comparison, the main lobe BWs, MSR, and PGL of all the SRA pairs in Figure 10 (except for TRA(2^2^,1^2^)/RRA(4^2^,1^2^)) were measured and are listed in Table 1 in the ascending order of the number of the active elements. One can see that the TRA(2^2^,1^2^)/RRA(2^2^,1^2^) pair has the lowest MSR and the highest PGL. In the cases of CGL-free pairs, both the PGL and the BW_50_ decreased (i.e., improve) and the MSR increased (i.e., improves) as more elements were used; all the PGL values were lower than −40 dB except for the most sparse SRA pair TRA(2^2^,1^2^)/RRA(3^2^,1^2^).

Specifically, compared to the dense array pair, the second most sparse array pair, TRA(2^2^,1^2^)/RRA(4^2^,2^2^), used only one in four elements of a dense array on both transmission (140) and reception (135) but provided almost the same BWs as that of the dense array pair with a PGL of −43.6 dB. This was comparable to the PGL value (−46.4 dB) of the ADS-based array pair [5] using more elements for both transmission (211) and reception (210). It should also be noticed that the steered responses in Figure 10b showed the same characteristics as those of the non-steered ones.

### 4.4. Sparse Spiral Array

**Figure 11:**
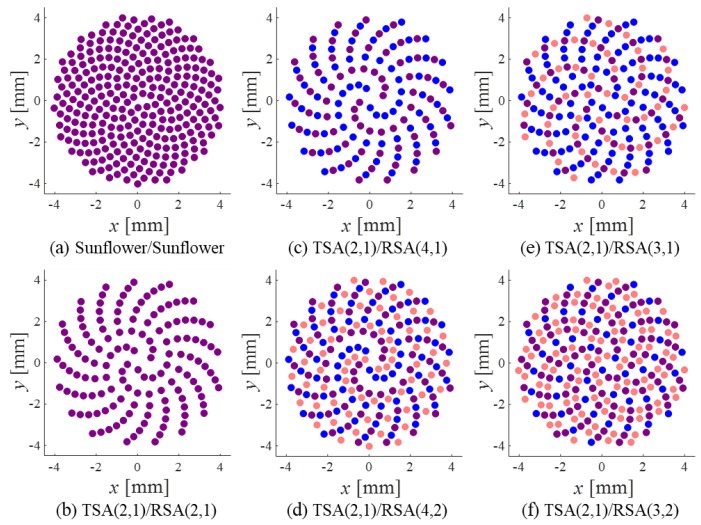
Transmit (blue) and receive (red) spiral array pairs: (**a**) the sunflower array pair and (**b**–**f**) the sparse spiral array (SSA) pairs. The overlapping elements are colored in purple.

Figure 11 shows a sunflower array pair and six different SSA pairs. Each array had a diameter of 16λ, the same as that of the SRAs in the previous subsection. The arrays were composed of circular elements, and each element had a diameter of $\left(1/\sqrt{\pi }\right)\lambda$ so that the element area was equal to that of the square element used for the SRAs. The dense array for the spiral arrays was the sunflower array shown in Figure 11a. The sunflower array is deliberately designed to have 280 elements so that the number of elements of SSA(2,1) is equal to that of SRA(2^2^,1^2^), which is the Tx array for each pair. Consequently, the sunflower array was designed to be much sparser than the dense rectangular array of 553 elements in the previous subsection. As in the SRA case, all SSA pairs in Figure 11b–f used the same TSA, TSA(2,1), and different RSAs. Among them, only the SSA pairs in Figure 11d–f followed the design rule presented in Section 3.

**Figure 12:**
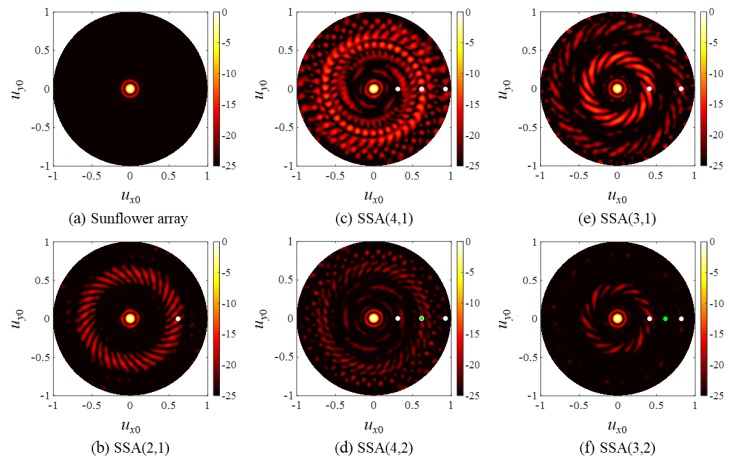
One-way, CW responses of (**a**) the sunflower array and (**b**–**f**) the SSAs. The radii of the grating lobe rings are indicated by white dots, and the radii of the nulling rings are indicated by green dots. A green dot in (d) is overlapped with the second white dot in the same plot.

The one-way CW responses of the sunflower array and the SSAs in Figure 11 are shown in Figure 12. Since the most likely interelement distance $\hat{d}$ of the sunflower array was calculated approximately as 0.81λ, according to Equation (20), the radius of its first grating lobe ring was expected to be λ/(1∙0.81λ) = 1.23. However, because this value exceeds unity, there will be no grating lobes in the field where $u_{x0}^{2}{\;+\;u}_{y0}^{2}\;<\;1$. This agrees with the simulated result shown in Figure 12a. For the SSAs, the radii of the grating lobe and the nulling rings were calculated by Equations (20) and (21), respectively, and their calculated values are listed in Table 2. The expected radii are represented by circular dots (white for grating lobe rings and green for nulling rings) in Figure 12.

For SSA(2,1) and SSA(3,1) (Figure 12b,e), the white dots represent the individual grating lobe rings well. For SSA(3,2) (Figure 12f), a single nulling ring is assumed to be present at the expected radial distance marked with a green dot. As the nulling ring served as a weighting function for the entire field, the first grating lobe ring of SSA(3,1) became much thinner and smaller in the beam pattern of SSA(3,2). Furthermore, the second grating lobe ring of SSA(3,2) and the grating lobes near the nulling ring is hardly observed in Figure 12f because of the nulling ring.

On the other hand, the grating lobe rings of SSA(4,1) in Figure 12c do not appear as distinctively as those of SSA(2,1) and SSA(3,1). Nonetheless, one can see that high amplitude grating lobes were present around the rings with the radii marked by the white dots in Figure 12c. As with the SSA(3,2), it is shown in Figure 12d that the second grating lobe ring of SSA(4,1) in Figure 12c was drastically suppressed in Figure 12d. The first and third grating lobe rings were also much suppressed. This is again due to the presence of the nulling ring marked with a green dot, which in this case, overlapped the second white dot in Figure 12d.

**Figure 13:**
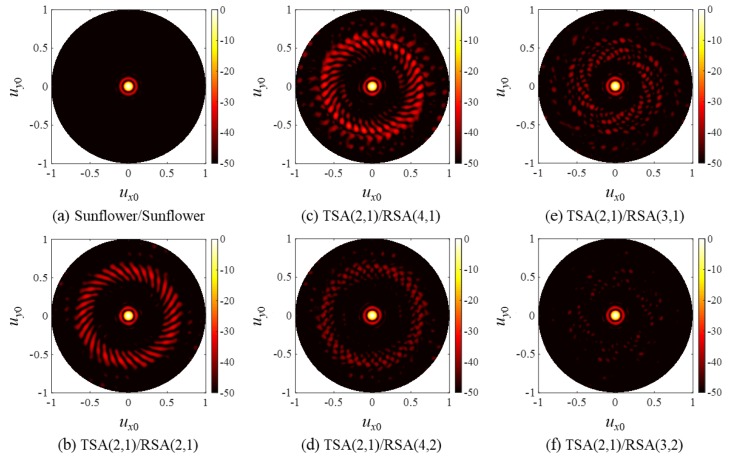
Two-way, CW PSFs of (**a**) the sunflower array pair and (**b**–**f**) the SSA pairs.

The two-way CW PSFs of the SSA pairs are shown in Figure 13. Because TSA(2,1)/RSA(4,2), TSA(2,1)/RSA(3,1), and TSA(2,1)/RSA(3,2) followed the design rule to avoid strong CGLs, their two-way PSFs had very low grating lobes, as shown in Figure 13d–f. As expected, the TSA(2,1)/RSA(3,2) pair had the smallest grating lobes among them. However, as shown in Figure 13b,c, TSA(2,1)/RSA(2,1) and TSA(2,1)/RSA(4,1) produced high grating lobes because of the presence of high CGLs around *r* = 0.62. Note that even though the TSA(2,1)/RSA(4,2) pair used the same number of elements as that of the TSA(2,1)/RSA(2,1) pair, its PSF had much lower grating lobes.

**Figure 14:**
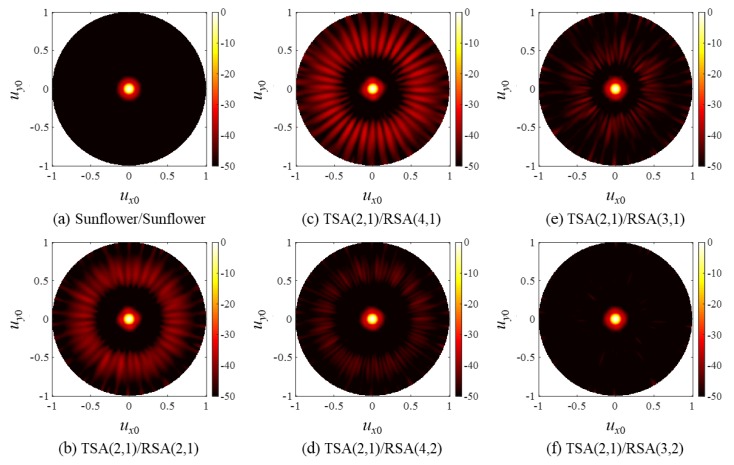
Two-way, PW PSFs of (**a**) the sunflower array pair and (**b**–**f**) the SSA pairs.

**Figure 15:**
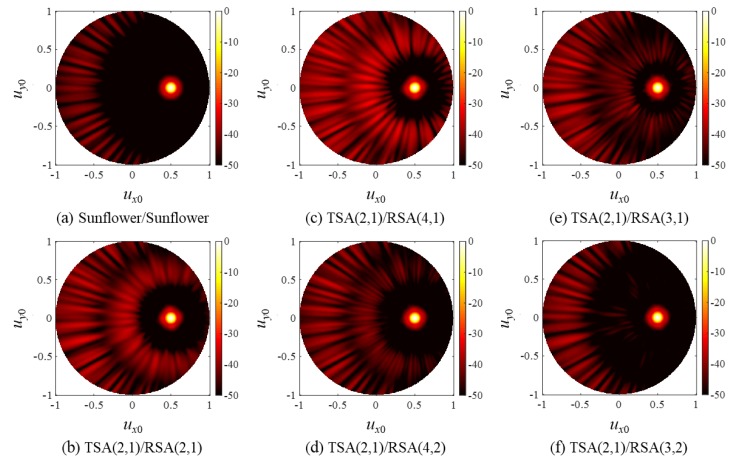
PW PSFs of (**a**) the sunflower array pair and (**b**–**f**) the SSA pairs for a steered focal point at (*u_x_*_f_,*u_y_*_f_) = (0.5,0) (i.e., θ_f_ = π/6, and φ_f_ = 0).

As in the cases of SRA pairs, the PW PSFs of the SSA pairs in Figure 14 agree well with the CW simulation results in Figure 13 except that they had lower but broader grating lobes, and the steered responses of the SSA pairs in Figure 15 also exhibited grating lobes near *u_x_*_0_ = −1. Note, however, that the grating lobe level near *u_x_*_0_ = −1 was smaller than that of the SRA pairs in Figure 9. This is because the elements of the spiral arrays were distributed irregularly, thereby spreading the grating lobe energy along all directions, whereas the elements of the rectangular arrays were placed regularly in specific directions (*x* and *y*).

**Figure 16:**
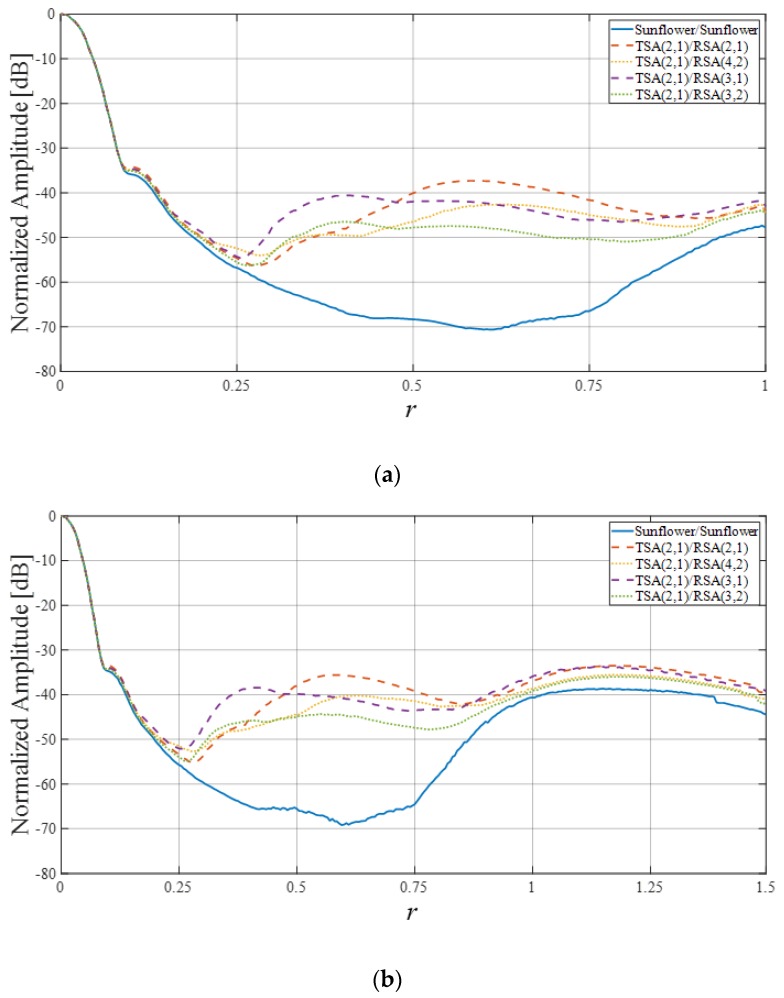
Peak 1D profiles of (**a**) non-steered and (**b**) steered PW PSFs of the spiral array pairs. For each *r*, the maximum value of the PSFs on the *r*-radius circle centered at the focal point is plotted. The steered focal point was set to (*u_x_*_f_,*u_y_*_f_) = (0.5,0) (i.e., θ_f_ = π/6, and φ_f_ = 0).

Figure 16 shows the 1D peak profiles of the non-steered and the steered PSFs of the spiral array pairs, where the horizontal axis represents the radius, $r^{2}\;=\;{\left(u_{x0}-u_{xf}\right)}^{2}\;+\;{\left(u_{x0}-u_{xf}\right)}^{2}$, of a circle centered at the focal point (*u_x_*_f_, *u_y_*_f_). For each *r*, the maximum value on the circle is plotted as the grating lobes of the spiral arrays had irregular circular patterns. For a quantitative comparison, the performance measures of the SSA pairs are listed in Table 3. The array pair TSA(2,1)/RSA(4,1) was excluded for comparison.

The sunflower array pair (Figure 11a) had the best performance with the highest MSR of 29.1 dB, lowest PGL of –47.2 dB, and the narrowest BW_50_ of 20.22°. All the SSA pairs that followed the design rule had PGL values below −40 dB, whereas TSA(2,1)/RSA(2,1) had a PGL of −37.3 dB even though it used the same number of elements as TSA(2,1)/RSA(4,2). Among the SSA pairs that follow the rule, the TSA(2,1)/RSA(3,2) pair, which used the largest number of active elements, had the best MSR (22.4 dB), PGL (−43.9 dB), and BW_50_ (20.68°). Just like the SRA pairs, Table 3 shows that the SSA pairs that followed the design rule had better MSR, PGL, and BW_50_ values when more active elements were used.

## 5. Discussion

In this paper, it was demonstrated that the proposed method to avoid common grating lobes in the Tx/Rx sparse array pairs can be effectively used in designing two types of sparse 2D array pairs: SRA and SSA pairs. As the rectangular arrays and the spiral arrays are the most widely used 2D arrays in both real imaging and experimental studies, the two sparse array schemes can be readily implemented on either commercially available arrays or the custom-made ones, which, in fact, was the primary purpose of this work. Hence, comparative evaluation of the beamforming characteristics of SRA and SSA pairs was not necessarily required. When newly fabricating a 2D array, however, one should decide which type of sparse arrays better suits its purpose.

The performances of the SRA and SSA pairs can be compared with the results summarized in Table 1 and Table 3. However, the number of elements of the two schemes were different. This is because the number of elements of each dense array (553 for rectangular array and 280 for sunflower array) was determined so that the transmit sparse arrays, TRA(2^2^,1) and TSA(2,1), should have the same number of active elements (140 elements). Consequently, the Rx arrays for the rectangular and the spiral array pairs had a different number of elements, as listed in Table 1 and Table 3. Under this condition, one can only expect to compare the relative characteristics of the two array types. Moreover, the element size (area) of the spiral arrays was chosen to be the same as that of the rectangular arrays. Because the sunflower array had fewer elements than the dense rectangular array, larger elements could be used for the spiral arrays.

**Figure 17:**
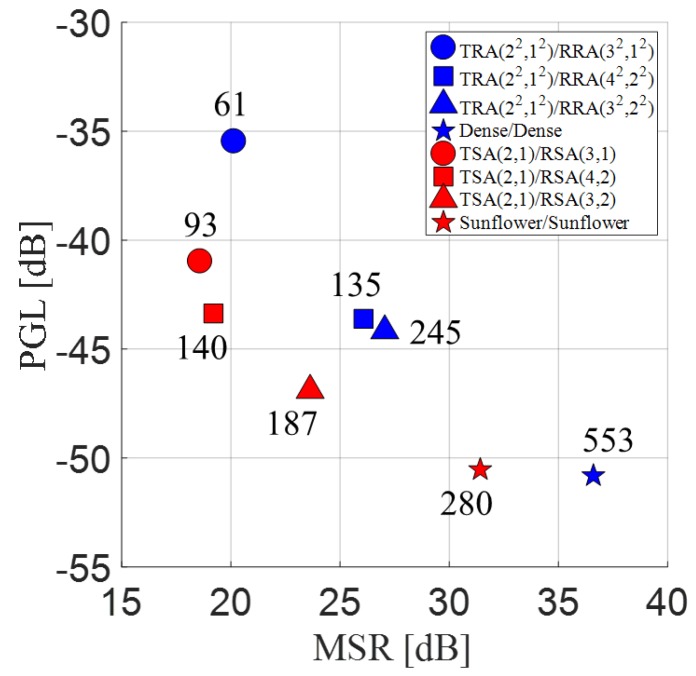
Main lobe-to-side lobe energy ratio (MSR) vs. peak grating lobe level (PGL) for the rectangular array pairs (blue) and the spiral array pairs (red). The number of elements of the receive array for each array pair is also marked. The same number of elements (140) were used in the transmit arrays for the sparse array pairs.

Here, for a fairer comparison, the element size of each array type was maximized independently, but under the same criterion of using the same kerf of 0.05λ between the adjacent elements. Consequently, the element diameter of the spiral arrays in Table 3 was increased to 0.67λ from 0.56λ, because the minimum interelement distance of the sunflower array was 0.77λ. With the enlarged elements for the spiral arrays, the performance of the array pairs improved by 1.0–2.3 dB in MSR, 0.4–3.3 dB in PGL, and 5.8 dB in main lobe peak intensity. Little effect on the main lobe BW was observed. The MSR and PGL values for the rectangular array pairs and the modified spiral array pairs are plotted in Figure 17, where each group of the sparse rectangular (blue) and the spiral (red) array pairs with the same *P* and *L* is represented by a unique shape.

The dense rectangular array (blue star) had the best performance in both the MSR (36.6 dB) and PGL (−50.8 dB) values. Although the sunflower array (red star) used nearly half the elements of the dense rectangular array (blue star), its PGL was −50.5 dB. The two array pairs perform almost the same in terms of PGL. But, the MSR (31.4 dB) of the sunflower array was smaller by about 5.2 dB than that of the dense rectangular array.

For the sparse arrays pairs, the SSA pairs had lower (better) PGL values than the SRA pairs except in one group with *P*_R_ = 4 and *L*_R_ = 2, while all the SRA pairs had higher (better) MSR values. These results can be explained by the fact that the periodic arrangement of elements in the rectangular arrays concentrates the grating lobe energy in certain directions, as was observed in Figure 7, Figure 8 and Figure 9, whereas the irregular spiral arrays spread the grating lobe energy, as shown in Figure 13, Figure 14 and Figure 15. Finally, it can be seen from Table 1 and Table 3 that the SSA pairs had narrower main lobe BWs (both BW_6_ and BW_50_) than the corresponding SRA pairs. As an exception mentioned above, the PGL of TSA(2,1)/RSA(4,2) (red square) was measured to be −43.4 dB, which was slightly higher than that (−43.6 dB) of TRA(2^2^,1^2^)/RRA(4^2^,2^2^) (blue square). We think this is because SSA(4,1) with only 70 elements was too sparse to produce the theoretically expected grating lobe and nulling rings. This effect on the simulated PSFs was investigated with Figure 12c,d.

The proposed design rule is very simple, effective, and readily applicable to commercially available or custom-made rectangular and sunflower arrays. As well as the dense rectangular array that has a perfectly uniform distribution of elements, the sunflower array is known to have the most uniform distribution of elements among the irregular arrays. The sparse array designs of both the SRAs and the SSAs conserve the uniformity of the corresponding dense arrays. Such arrays with uniform element distributions offer significant advantages: (1) The element size can be maximized to obtain the highest SNR; (2) uniform beam patterns can be obtained along any scanning direction, and (3) uniform 2D arrays are relatively easy to fabricate.

So far, spiral arrays have been studied mainly to reduce the huge number of elements of rectangular arrays [14,15]. However, to the best of the authors’ knowledge, no attempt on designing Tx/Rx sparse spiral array pairs on a sunflower array has been made. In this paper, we have shown that the number of active elements of the spiral arrays can be further reduced by the proposed design rule when necessary. The SSA pairs, which have advantages of lower PGL and narrower BW compared to the SRA pairs, may find increasing application as a cost-effective method of manufacturing spiral arrays has recently been reported [23].

Further improvement in the SRA pairs can be achieved by optimizing the positions of *L_x_*∙*L_y_* active elements in the subarray block of *P_x_*∙*P_y_* elements rather than fixing the positions, as shown in Figure 2. We think there also exist other approaches for designing more efficient SSA pairs, for example, using various optimization techniques. The density-tapered spiral arrays [15] can also be adapted to the proposed SSAs to improve the imaging quality. We also plan to curve the proposed sparse array designs as the curving improves beamforming performances [24].

## 6. Conclusions

Sparse 2D array designs, SRAs, and SSAs, based on a rectangular and a sunflower gird, respectively, were presented. Design rules for the optimal Tx/Rx pair of each of the SRAs and the SSAs were also developed from the structured models of the arrays. The purpose of the design rule was to pair the Tx/Rx sparse arrays without any CGLs in their beam patterns. The rules were developed by the theoretical models for the beam patterns of the arrays. The verification of the design rules for both schemes was done by assessing the simulated field responses. The results showed that the sparse array pairs following the design rule have PSFs of much-suppressed grating lobes than the ones that did not follow the rule while maintaining similar BWs of that of the dense array pair.

The implementation of the proposed sparse array pairs is not difficult due to the commercially available arrays and the advanced manufacturing technology for 2D arrays. Thus, the experimental evaluation of the sparse array pairs is ready to be done. Although comparing the beamforming performance of the two array schemes was not the main purpose for this work, we have also discussed the pros and cons of the two schemes for the future development of a new 3D imaging probe. The comparison study showed that the SRA pairs had higher MSR than the SSA pairs, and the SSA pairs had advantages on low PGLs and narrow BWs.

Previous design methods for sparse 2D arrays either lacked array uniformity or generalization for the design rule, and they were mostly based on rectangular arrays. A few studies emphasized the good beamforming performance of the spiral arrays, but various ways of designing Tx/Rx sparse array pairs were yet to be studied intensively. This paper presents a generalized design rule for the SRA array pairs that can be easily implemented on the existing 2D array probes and paves a way of developing further improved sparse spiral array designs.

## Figures and Tables

**Table 1:** Properties of near-field, pulsed-wave responses of rectangular array pairs.

Figure	Tx		Rx		BW [deg]		MSR [dB]	PGL [dB]
	Design	# of Elements	Design	# of Elements	BW_6_	BW_50_		
Figure 5e	TRA(2^2^,1^2^)	140	RRA(3^2^,1^2^)	61	3.84	24.60	20.1	−35.5
Figure 5d	TRA(2^2^,1^2^)	140	RRA(4^2^,2^2^)	135	3.84	22.96	26.1	−43.6
Figure 5b	TRA(2^2^,1^2^)	140	RRA(2^2^,1^2^)	140	3.78	24.77	1.6	−14.3
Figure 5f	TRA(2^2^,1^2^)	140	RRA(3^2^,2^2^)	245	3.84	22.49	27.0	−44.2
Figure 5a	Dense	553	Dense	553	3.84	20.80	36.6	−50.8

**Table 2:** Expected radii of the grating lobe rings and the nulling rings for the SSAs.

SSA	Radii of the Grating Lobe Rings (*r*_gl_)	Radii of the Nulling Rings (*r*_null_)
SSA(2,1)	0.62	1.23 (>1)
SSA(4,1)	0.31, 0.62, 0.93	1.23 (>1)
SSA(4,2)	0.31, 0.62, 0.93	0.62, 1.23 (>1)
SSA(3,1)	0.41, 0.82	1.23 (>1)
SSA(3,2)	0.41, 0.82	0.62, 1.23 (>1)

**Table 3:** Properties of near-field, pulsed-wave responses of spiral array pairs.

Figure	Tx		Rx		BW [deg]		MSR [dB]	PGL [dB]
	Design	# of Elements	Design	# of Elements	BW_6_	BW_50_		
Figure 11e	TSA(2,1)	140	RSA(3,1)	93	3.78	20.91	17.6	−40.6
Figure 11d	TSA(2,1)	140	RSA(4,2)	140	3.84	21.44	18.1	−42.4
Figure 11b	TSA(2,1)	140	RSA(2,1)	140	3.78	21.09	14.5	−37.3
Figure 11f	TSA(2,1)	140	RSA(3,2)	187	3.78	20.68	22.4	−43.9
Figure 11a	Sunflower	280	Sunflower	280	3.78	20.22	29.1	−47.2

## References

[1] Boni C., Richard M., Barbarossa S. Optimal configuration and weighting of nonuniform arrays according to a maximum ISLR criterion. Proceedings of the IEEE International Conference on Acoustics, Speech and Signal Processing.

[2] Choe J.W., Oralkan Ö., Khuri-Yakub P.T. Design optimization for a 2-D sparse transducer array for 3-D ultrasound imaging. Proceedings of the IEEE Ultrasonics Symposium.

[3] Diarra B., Robini M., Tortoli P., Cachard C., Liebgott H. (2013). Design of optimal 2-D nongrid sparse arrays for medical ultrasound. IEEE Trans. Biomed. Eng..

[4] Roux E., Varray F., Petrusca L., Cachard C., Tortoli P., Liebgott H. (2018). Experimental 3-D ultrasound imaging with 2-D sparse arrays using focused and diverging waves. Sci. Rep..

[5] Oliveri G., Massa A. (2010). ADS-based array design for 2-D and 3-D ultrasound imaging. IEEE Trans. Ultrason. Ferroelectr. Freq. Control.

[6] Lockwood G.R., Li P.C., O’Donnell M., Foster F.S. (1996). Optimizing the radiation pattern of sparse periodic linear arrays. IEEE Trans. Ultrason. Ferroelectr. Freq. Control.

[7] Nikolov S.I., Jensen J.A. (2000). Application of different spatial sampling patterns for sparse array transducer design. Ultrasonics.

[8] Austeng A., Holm S. (2002). Sparse 2-D arrays for 3-D phased array imaging—Design methods. IEEE Trans. Ultrason. Ferroelectr. Freq. Control.

[9] Austeng A., Holm S. (2002). Sparse 2-D arrays for 3-D phased array imaging—Experimental validation. IEEE Trans. Ultrason. Ferroelectr. Freq. Control.

[10] Song J.H., Lee J., Yeo S., Kim G.-D., Song T.-K. (2019). An analytical approach to designing optimal sparse 1D phased arrays for handheld ultrasound imaging. IEEE Trans. Ultrason. Ferroelectr. Freq. Control.

[11] Wang Y., Stephens D.N., O’donnell M. (2002). Optimizing the beam pattern of a forward-viewing ring-annular ultrasound array for intravascular imaging. IEEE Trans. Ultrason. Ferroelectr. Freq. Control.

[12] Ullate L.G., Godoy G., Martínez O., Sánchez T. (2006). Beam steering with segmented annular arrays. IEEE Trans. Ultrason. Ferroelectr. Freq. Control.

[13] Schwartz J.L., Steinberg B.D. (1998). Ultrasparse, ultrawideband arrays. IEEE Trans. Ultrason. Ferroelectr. Freq. Control.

[14] Martínez-Graullera O., Martín C.J., Godoy G., Ullate L.G. (2010). 2D array design based on Fermat spiral for ultrasound imaging. Ultrasonics.

[15] Ramalli A., Boni E., Savoia A.S., Tortoli P. (2015). Density-tapered spiral arrays for ultrasound 3-D imaging. IEEE Trans. Ultrason. Ferroelectr. Freq. Control.

[16] Macovski A. (1979). Ultrasonic imaging using arrays. Proc. IEEE.

[17] Bae S., Park J., Song T.-K. (2019). Contrast and volume rate enhancement of 3D ultrasound imaging using aperiodic plane wave angles: A simulation study. IEEE Trans. Ultrason. Ferroelectr. Freq. Control.

[18] Liew S.F., Noh H., Trevino J., Negro L.D., Cao H. (2011). Localized photonic band edge modes and orbital angular momenta of light in a golden-angle spiral. Opt. Express.

[19] Jensen J.A., Svendsen N.B. (1992). Calculation of pressure fields from arbitrarily shaped, apodized, and excited ultrasound transducers. IEEE Trans. Ultrason. Ferroelectr. Freq. Control.

[20] Jensen J.A. (1996). Field: A program for simulating ultrasound systems. Med. Biol. Eng. Comput..

[21] Turnbull D.H., Foster F.S. (1991). Beam steering with pulsed two-dimensional transducer arrays. IEEE Trans. Ultrason. Ferroelectr. Freq. Control.

[22] Weight J.P. (1984). Ultrasonic beam structures in fluid media. J. Acoust. Soc. Am..

[23] Martínez-Graullera O., Yagüe-Jiménez V., Paetsch A.B., Rodríguez A.I. The role of additive manufacturing technology in the design of sparse transducer arrays. Proceedings of the IEEE Ultrasonics Symposium.

[24] Kirkebø J.E., Austeng A. (2007). Improved beamforming using curved sparse 2D arrays in ultrasound. Ultrasonics.

